# The molybdate-binding protein ModA is required for *Proteus mirabilis*-induced UTI

**DOI:** 10.3389/fmicb.2023.1156273

**Published:** 2023-04-27

**Authors:** Yi Huang, Jinbin Chen, Qiao Jiang, Nan Huang, Xin Ding, Liang Peng, Xiaoyan Deng

**Affiliations:** ^1^Guangzhou Key Laboratory for Clinical Rapid Diagnosis and Early Warning of Infectious Diseases, Guangzhou Medical University, Guangzhou, Guangdong, China; ^2^KingMed School of Laboratory Medicine, Guangzhou Medical University, Guangzhou, Guangdong, China; ^3^Guangdong 999 Brain Hospital, Guangzhou, Guangdong, China; ^4^The Fifth Affiliated Hospital of Guangzhou Medical University, Guangzhou, Guangdong, China

**Keywords:** molybdate transport, *Proteus mirabilis*, urinary tract infection, transcriptomic, pathogenicity

## Abstract

**Background:**

*Proteus mirabilis* is one of the pathogens commonly causing urinary tract infections (UTIs). The molybdate-binding protein ModA encoded by *modA* binds molybdate with high affinity and transports it. Increasing evidence shows that ModA promotes the survival of bacteria in anaerobic environments and participates in bacterial virulence by obtaining molybdenum. However, the role of ModA in the pathogenesis of *P. mirabilis* remains unknown.

**Results:**

In this study, a series of phenotypic assays and transcriptomic analyses were used to study the role of ModA in the UTIs induced by *P. mirabilis*. Our data showed that ModA absorbed molybdate with high affinity and incorporated it into molybdopterin, thus affecting the anaerobic growth of *P. mirabilis*. Loss of ModA enhanced bacterial swarming and swimming and up-regulated the expression of multiple genes in flagellar assembly pathway. The loss of ModA also resulted in decreased biofilm formation under anaerobic growth conditions. The *modA* mutant significantly inhibited bacterial adhesion and invasion to urinary tract epithelial cells and down-regulated the expression of multiple genes associated with pilus assembly. Those alterations were not due to anaerobic growth defects. In addition, the decreased bacteria in the bladder tissue, the weakened inflammatory damage, the low level of IL-6, and minor weight change was observed in the UTI mouse model infected with *modA* mutant.

**Conclusion:**

Here, we reported that in *P. mirabilis*, ModA mediated the transport of molybdate, thereby affecting the activity of nitrate reductase and thus affecting the growth of bacteria under anaerobic conditions. Overall, this study clarified the indirect role of ModA in the anaerobic growth, motility, biofilm formation, and pathogenicity of *P. mirabilis* and its possible pathway, and emphasized the importance of the molybdate-binding protein ModA to *P. mirabilis* in mediating molybdate uptake, allowing the bacterium to adapt to complex environmental conditions and cause UTIs. Our results provided valuable information on the pathogenesis of ModA-induced *P. mirabilis* UTIs and may facilitate the development of new treatment strategies.

## Introduction

Urinary tract infections (UTIs) are among the commonest bacterial infectious diseases in humans and often occur in both community and hospital settings. Globally, UTIs account for about 40% of all hospital-acquired infections annually, and therefore constitute a major healthcare burden (Nicolle, 2008; Totsika et al., 2011). Among these infections, catheter-associated UTIs are the commonest hospital infections, primarily because bacteria can establish biofilms in catheters and thus resist clearance by the host defense system or antibiotics (Foxman, 2010; Lo et al., 2014). The production of adhesins, together with toxins, allows many pathogens to move to and colonize the bladder, causing UTIs (Alamuri et al., 2010). These infections can be either asymptomatic or symptomatic, with a wide spectrum of symptoms (Becknell et al., 2015). The commonest manifestation is acute cystitis, which can lead to bacteremia, sepsis, and even fatal consequences in severe cases (McLellan and Hunstad, 2016). Because there is an alarming increase in the rate of antimicrobial resistance (AMR) among uropathogens, the acute infection does not rule out the possibility of recurrence, which may constitute both an economic burden and a unique challenge to therapeutic antibiotic strategies (Hrbacek et al., 2020).

*Proteus mirabilis*, most noted for its ability to swarm across an agar plate, is one of the pathogens most frequently responsible for UTIs (Mobley, 2019). In the process of swarming, the bacterium changes from a short bacillus to a highly elongated, highly flagellated form, which clusters in crowds to move across the agar (Armbruster et al., 2018). *Proteus mirabilis* also produces abundant adhesins, which mediate its adhesion through the synthesis of pili (Pearson et al., 2008). All these characteristics are ultimately related to the pathogenesis and molecular physiology of *P. mirabilis*. However, the molecular mechanisms underlying the actions of *P. mirabilis* remain unclear. The infection of urethral catheters has been widely studied (Armbruster et al., 2018). Affected patients are susceptible to the formation of stones that can block the urinary tract (Mobley, 2019). Moreover, the consequences of the formation of *P. mirabilis* biofilms should not be underestimated. The anaerobic environment formed by a biofilm and the nitrate environment of the urethra mediate the anaerobic respiration of *P. mirabilis* and also protect the pathogen from elimination by the host’s immune system or antibiotics (Armbruster et al., 2018; Yu et al., 2021). The unknown pathogenic mechanisms of *P. mirabilis*, combined with the increase in AMR in recent years, warrant further investigation.

The *modA* gene encodes the molybdate-binding protein ModA, which binds with high affinity to the trace element molybdenum for its transportation. ModA has fascinated researchers, who noted that molybdenum binds tightly to ModA only at nanomolar concentrations (Lopez et al., 1993; Imperial et al., 1998; Ge et al., 2020). The molybdate (MoO_4_^2−^) transported into bacteria is incorporated into the molybdopterin precursor before the synthesis of molybdoenzyme, and then participates in the anaerobic respiration of the bacterium (Hoffmann et al., 2014). The *modA* gene has been identified in a variety of bacteria (Demtroder et al., 2019), and the involvement of ModA in virulence is well documented. Recent studies have focused on the role of ModA in bacterial virulence and pathogenicity, and many scholars have reported that it plays a key role in muscle infections caused by *Klebsiella pneumoniae*, chronic pulmonary infections caused by *Pseudomonas aeruginosa* (*Ps. aeruginosa*), pulmonary infections caused by *Mycobacterium tuberculosis*, and intestinal inflammation caused by Enterobacteriaceae (Camacho et al., 1999; Filiatrault et al., 2013; Williams et al., 2014; Perinet et al., 2016; Levillain et al., 2017; Zhu et al., 2018). However, no studies have shown the involvement of ModA in the virulence of *P. mirabilis*. Given the importance of ModA in virulence, the virulence mechanism of ModA in *P. mirabilis* warrants investigation to better understand how *P. mirabilis* causes UTIs.

We previously isolated a *P. mirabilis* strain without swarming motility from patients with clinical UTI, and an analysis of its transcriptome showed the significantly upregulated expression of its *modA* gene (Peng et al., 2020). Therefore, we speculated that ModA played an important role in the UTIs caused by *P. mirabilis*. We performed a series of experiments related to bacterial material transport, growth, motility, and pathogenicity to determine the relationship between ModA and the swarming ability of *P. mirabilis* and the effect of ModA on the UTIs caused by *P. mirabilis*. We used *P. mirabilis* strain DP2019 as the reference strain for high-throughput sequencing and an alignment analysis of the transcriptomes of *ΔmodA* mutant strain. Reverse transcription-quantitative PCR (RT-qPCR) was used to compare and verify the genes differentially expressed in this mutant. In this study, we comprehensively analyzed the effect of *modA* gene knockout on the biological processes of *P. mirabilis* that play crucial roles in molybdate transport, anaerobic growth, motility, and virulence, to clarify the mechanisms of ModA in UTIs caused by *P. mirabilis*.

## Results

### ModA affects the bacterial transport of molybdate to synthesize molybdoenzymes and participate in anaerobic respiration

**Figure 1:**
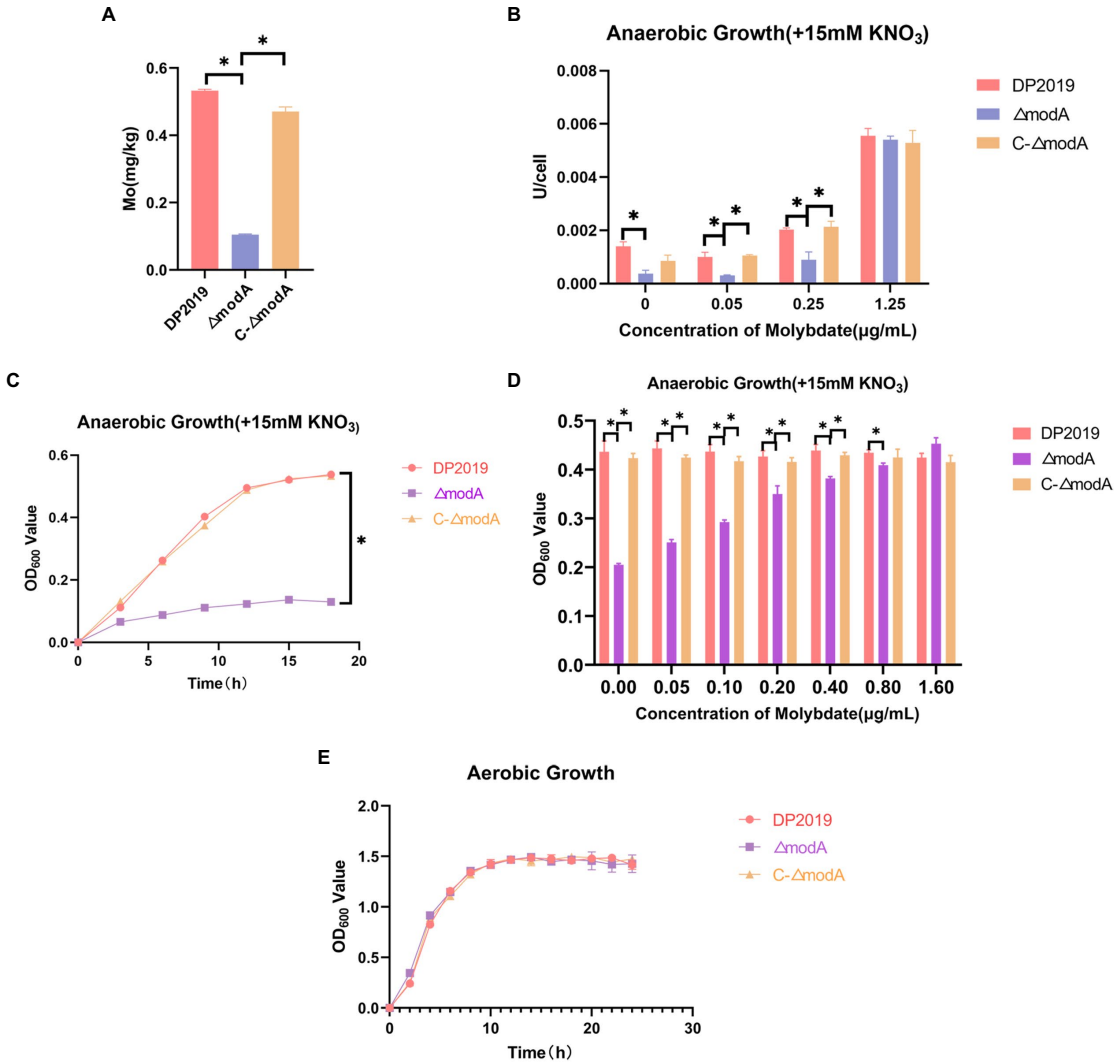
ModA affects bacterial transport of molybdate to synthesize molybdoenzymes and thus participates in anaerobic respiration. **(A)** Bacterial intracellular molybdenum content. **(B)** Under anaerobic conditions, 15 mM KNO_3_ was added to the culture medium, and the nitrate reductase activity was measured at different molybdate concentrations. **(C)** Anaerobic growth curve of bacteria when 15 mM KNO_3_ was added to the culture medium. **(D)** Anaerobic growth for 12h at different molybdate concentrations in medium supplemented with 15 mM KNO_3_. **(E)** Aerobic growth curve of bacteria after the addition of 15 mM KNO_3_ to the culture medium. Data are presented as the mean ± S.D. of three independent experiments, *p < 0.5.

The periplasmic binding protein encoded by *modA* plays an important role in the high-affinity transport of molybdenum. The content of molybdenum was measured with inductively coupled plasma mass spectrometry (ICP-MS) to determine whether bacteria with a defective *modA* gene accumulate molybdenum. These cells accumulated less molybdenum than wild-type cells or the *ΔmodA*-complemented strain *C-ΔmodA*. Our data show that the intracellular molybdenum concentration was significantly reduced in *ΔmodA*, but was essentially the same in the complemented strain *C-ΔmodA* as in wild-type strain DP2019, indicating that knocking-out the *modA* gene affected the ability of *P. mirabilis* to transport molybdate (Figure 1A). After the bacterial intake of molybdate, it is incorporated into the molybdenum cofactor (MoCo), which is necessary for the synthesis of molybdoenzyme activity in the molybdopterin molecule (Kraft et al., 2011). Nitrate reductase is a typical molybdenum-containing enzyme that reduces nitrate to nitrite in the initial step of the anaerobic growth process, acting as the terminal oxidase in the anaerobic respiratory chain (Kraft et al., 2011). Under anaerobic conditions, the rate of nitrate reduction is reduced in *Ps. aeruginosa ΔmodA* mutant strain (Pederick et al., 2014). Because *Ps. aeruginosa* ModA is a high-affinity molybdate transporter, the inhibited activity of the *ΔmodA* mutant was reversed by a high concentration of molybdate in the culture medium (Pederick et al., 2014). *mod* mutations in *E. coli* was previously demonstrated to cause pleiotropic effects on molybdoenzyme activity, including nitrate reductase activity (Glaser and DeMoss, 1971). Therefore, under anaerobic conditions in the presence of nitrate, we measured the nitrate reductase activity of the *ΔmodA* mutant when different concentrations of molybdate were added to the medium. At low concentrations of molybdate, *ΔmodA* showed lower nitrate reductase activity than wild-type DP2019 under anaerobic conditions, whereas the nitrate reductase activity of the complemented strain *C-ΔmodA* was slightly restored to the wild-type level. When the environmental molybdate concentration was increased to approximately 1 μg/mL levels, the nitrate reductase activity of DP2019 and *C-ΔmodA* increased gradually, and the nitrate reductase activity of the mutant *ΔmodA* recovered to levels similar to those of DP2019, suggesting that the reduced molybdate availability in *modA* mutant results in poorer nitrate reductase activity (Figure 1B). It has also been reported that *modA* mutants of *Ps. aeruginosa* and *K. pneumoniae* show reduced nitrate reductase activity and anaerobic growth under anaerobic and nitrate-present conditions (Garzon et al., 1992; Perinet et al., 2016; Wang et al., 2021). The finding that ModA affects nitrate reductase activity prompted us to hypothesize that it may also be involved in anaerobic respiration. To this end, the growth rate of the *ΔmodA* mutant was evaluated under anaerobic conditions in the presence of nitrate. Under anaerobic conditions, the growth of the *ΔmodA* mutant in Luria-Bertani (LB) medium supplemented with 15 mM KNO_3_ was impaired compared with that of strain DP2019, and no growth difference has been noticed between DP2019 and the *ΔmodA* mutant (Figures 1C,E). We also tested the anaerobic growth of the bacteria when different concentrations of molybdenum were added to the medium. Our data showed that compared with DP2019, the anaerobic growth of *ΔmodA* was significantly inhibited, whereas this defect was reversed in *C-ΔmodA*. As the molybdenum concentration increased, the inhibition of the anaerobic growth of the *ΔmodA* mutant recovered slowly, but still differed significantly from those of DP2019 and *C-ΔmodA*. Interestingly, at a high molybdenum concentration of 1.6 μM, the anaerobic growth of *ΔmodA* was restored to the levels of DP2019 and *C-ΔmodA* (Figure 1D). These data indicated that ModA mediated the high-affinity transport of molybdenum by *P. mirabilis* at low molybdenum concentrations and that other transporters participated in molybdate transport at higher molybdenum concentrations, thus restoring the defect in the mutant *ΔmodA*. Therefore, ModA affected the high-affinity absorption of molybdate, thereby affecting the activity of nitrate reductase, which contained molybdate and was crucial for the survival of *P. mirabilis* in anaerobic environments.

### Loss of ModA resulted in increased swarming motility and swimming motility

**Figure 2:**
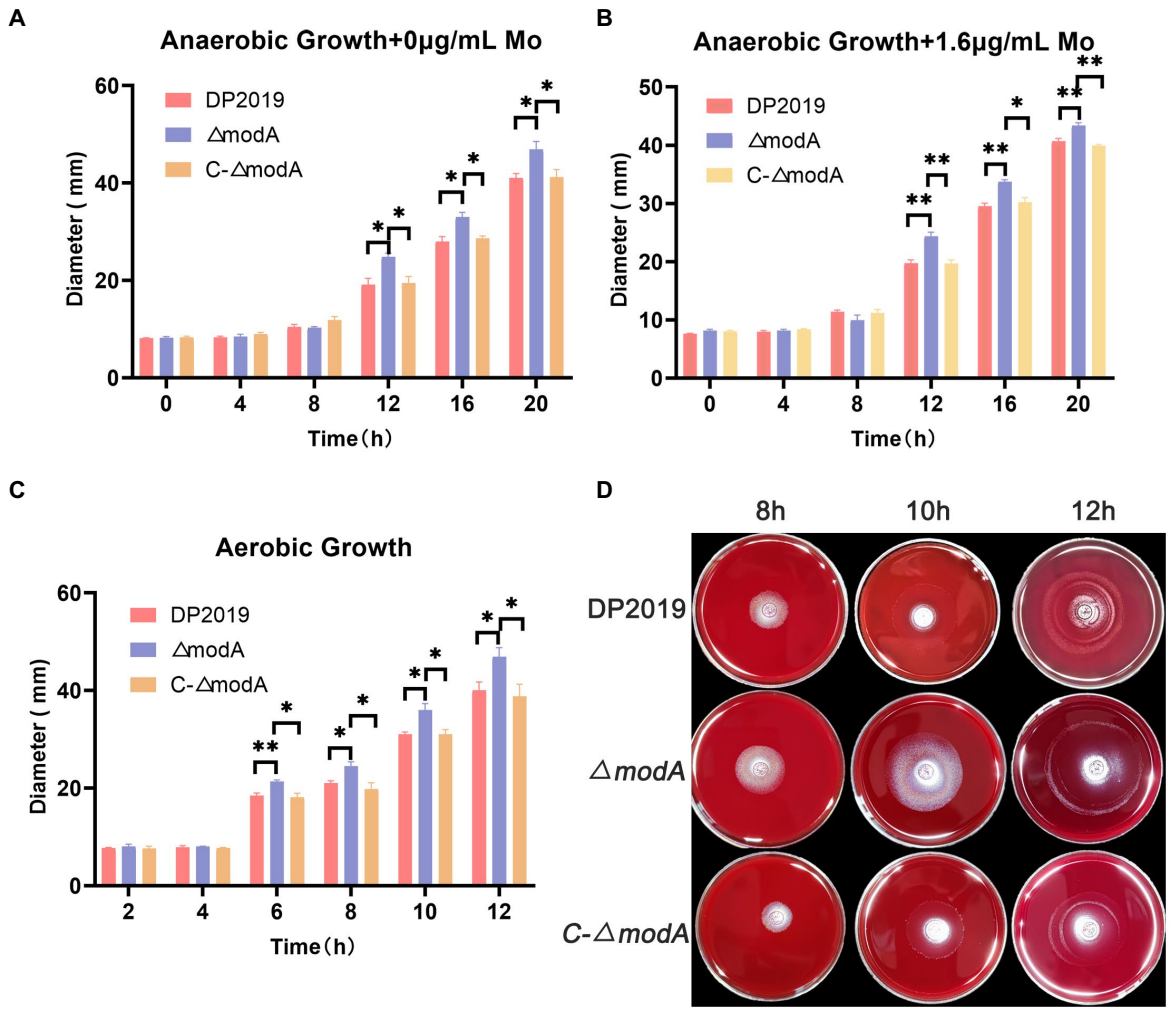
Examination of swarming motility. **(A)** Line graph showing swarming migration distances under anaerobic growth conditions. **(B)** Line graph showing swarming migration distances under anaerobic growth conditions with 1.6 μg/mL molybdate supplement to avoid the interference of anaerobic growth defects on the results. **(C)** Line graph showing swarming migration distances under aerobic growth conditions. **(D)** Halo images of bacteria swarming on blood agar plates under aerobic growth conditions. Data are presented as the mean ± S.D. of three independent experiments, *p < 0.5, **p < 0.01.

**Figure 3:**
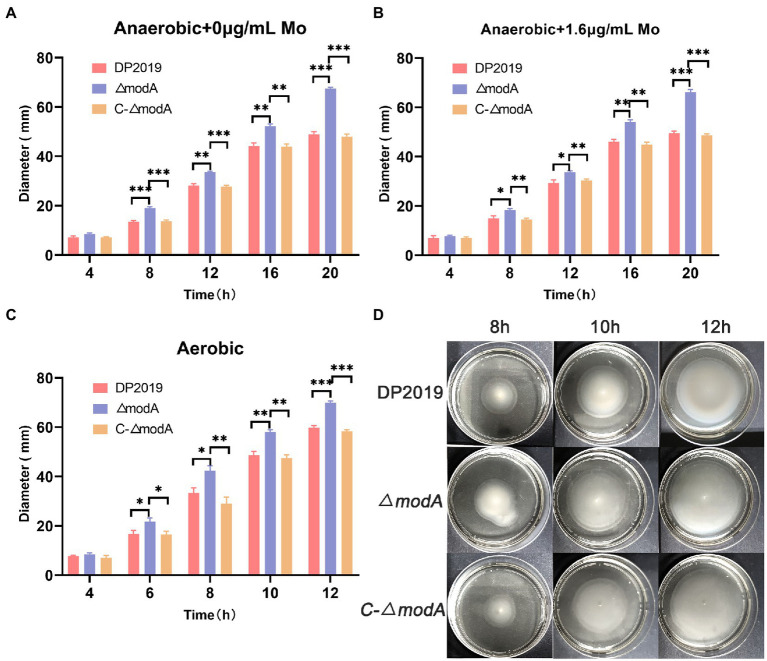
Examination of swimming motility. **(A)** Line graph showing swimming migration distances under anaerobic growth conditions. **(B)** Line graph showing swimming migration distances under anaerobic growth conditions with 1.6 μg/mL molybdate supplement to avoid the interference of anaerobic growth defects on the results. **(C)** Line graph showing swimming migration distances under aerobic growth conditions. **(D)** Halo images of bacteria swimming on agar plates under aerobic growth conditions. Data are presented as the mean ± S.D. of three independent experiments, *p < 0.5, **p < 0.01, ***p < 0.01.

A previous transcriptome of a *P. mirabilis* strain with swarming migration deficiency showed the significantly upregulated expression of its *modA* gene (Peng et al., 2020). To evaluate the effect of ModA on the motility of *P. mirabilis*, we performed a series of experiments to examine the motility of *P. mirabilis*, including swarming motility and swimming motility. Swarming motility and swimming motility can be distinguished according to solid and liquid media, respectively. Figures 2, 3 show that after the bacteria entered the logarithmic growth period, the motility characteristics of each strain varied tremendously. Under anaerobic growth conditions, at each time point after 12 h, the migration loop of strain *ΔmodA* was the largest among the strains compared, whereas the migration loop of the complemented strain *C-ΔmodA* was the same size as that of wild-type strain DP2019 (Figures 2A, 3A). Our previous anaerobic growth experiment showed that the growth defects of the mutant could be restored under the condition of high concentration molybdate (Figure 1B). We designed and experimented with the anaerobic growth condition of each strain in 1.6 μg/mL molybdate in to avoid the interference of anaerobic growth defects on the results. Results showed that the enhancement was independent of molybdate supplementation, which implies that the increased motility in *ΔmodA* is not related to anaerobic growth defects (Figures 2B, 3B). Since the previous swarming migration assay of clinical strains with up-regulated expression of the *modA* gene was carried out under aerobic conditions, we also tested the motility under aerobic conditions (Peng et al., 2020). Under aerobic conditions, the migration rate of each strain is faster than that under anaerobic conditions. At every 2 h after 6 h, the migration diameter of the *ΔmodA* strain is the largest (Figures 2C,D). Overall, these data demonstrated that ModA affected the swarming motility and swimming motility of *P. mirabilis*, which has nothing to do with the existence of oxygen, and the effect is not due to anaerobic growth defects.

### ModA is crucial to the formation of the *Proteus mirabilis* biofilm

**Figure 4:**
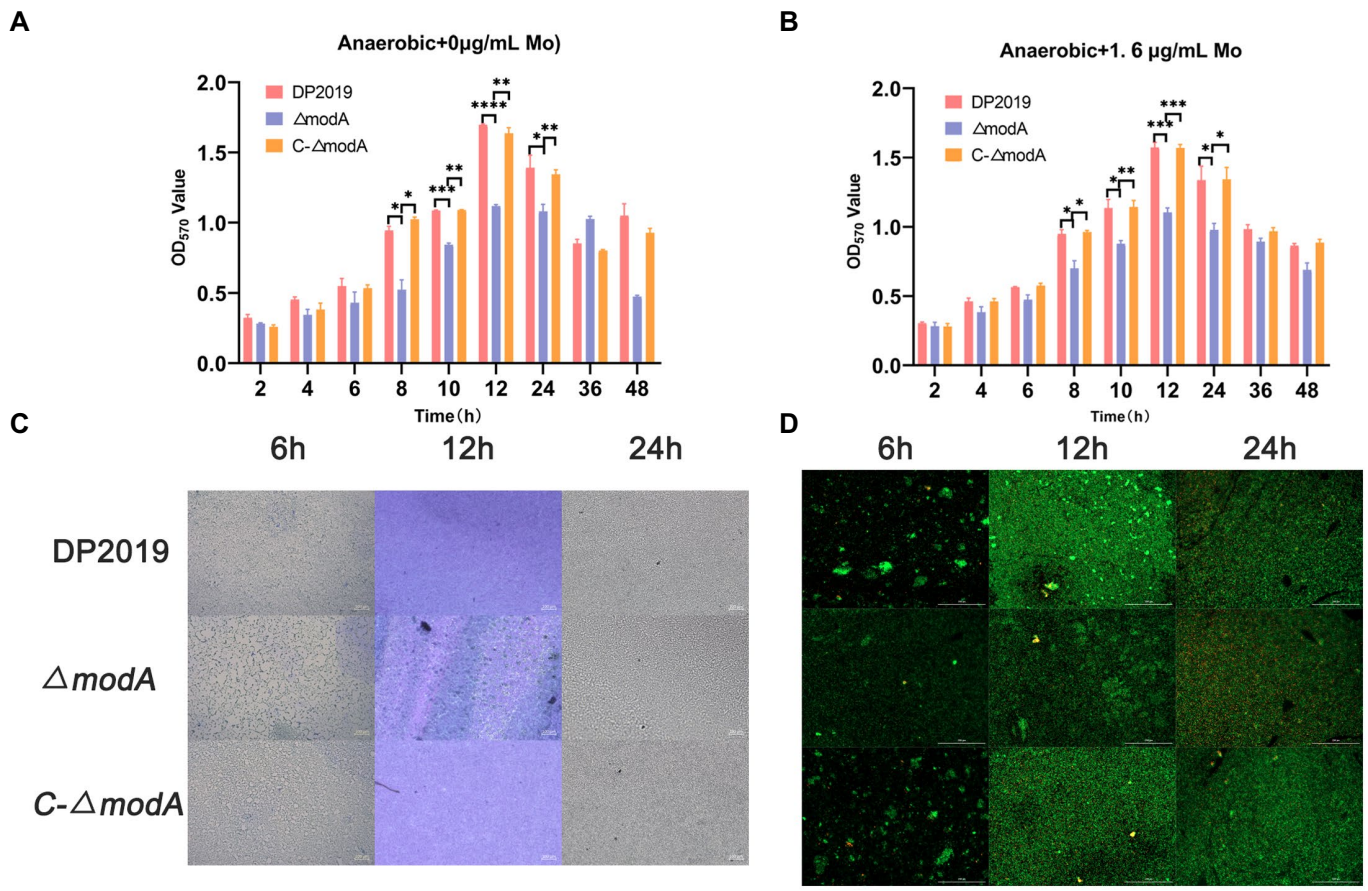
Detection of biofilm-forming ability. **(A)** Quantification of biofilm formation. Biofilm formation was quantified by measuring the absorbance at various time points after Crystal Violet staining under anaerobic growth conditions. The optical density (570 nm) of the solution extracted with ethanol/acetone correlated with the level of biofilm formation. **(B)** Quantification of biofilm formation. Biofilm formation was quantified by measuring the absorbance at various time points after Crystal Violet staining in 1.6 μg/mL molybdate under anaerobic growth conditions. **(C)** Images of biofilms stained with Crystal Violet at different time points. **(D)** The two-color fluorescence assay at various time points under anaerobic growth conditions. Data are presented as the mean ± S.D. of three independent experiments, *p < 0.5, **p < 0.1, ***p < 0.001, ****p < 0.0001.

Perinet et al. (2016) found that the decrease in biofilm formation observed in the *modA* mutant of *Ps. aeruginosa* was not due to its anaerobic growth defects. A crystal violet staining experiment showed that the bacterial biofilm formed gradually and became stable at 12 h, after which it began to disintegrate. The weak biofilm-forming ability of mutant strain *ΔmodA* was observed at each time point between 8 and 24 h, whereas the biofilm-forming ability of *C-ΔmodA* was restored to the wild-type level (Figure 4A). The crystal violet staining experiment and two-color fluorescence assay (LIVE/DEAD^®^ BacLight^™^ Bacterial Viability Kit, Molecular Probes) showed similar trends in biofilm formation at 6, 12, and 24 h. At 12 h, the wild-type DP2019 and *C-ΔmodA* strains formed thick and complete biofilms, whereas the biofilm of strain *ΔmodA* was less well formed and more broken. At 24 h, the biofilms of wild-type DP2019 and *C-ΔmodA* strains were thin but still complete, whereas that of *ΔmodA* was red, indicating the presence of dead bacteria (Figures 4C,D). We designed and experimented with the anaerobic growth condition of each strain in 1.6 μg/mL molybdate, which implies that reduced biofilm formation in *ΔmodA* is not related to its growth anaerobic growth defects (Figure 4B). These results demonstrate that ModA is crucial to the formation of the *P. mirabilis* biofilm.

### ModA is important for virulence in the cell model *in vitro*

**Figure 5:**
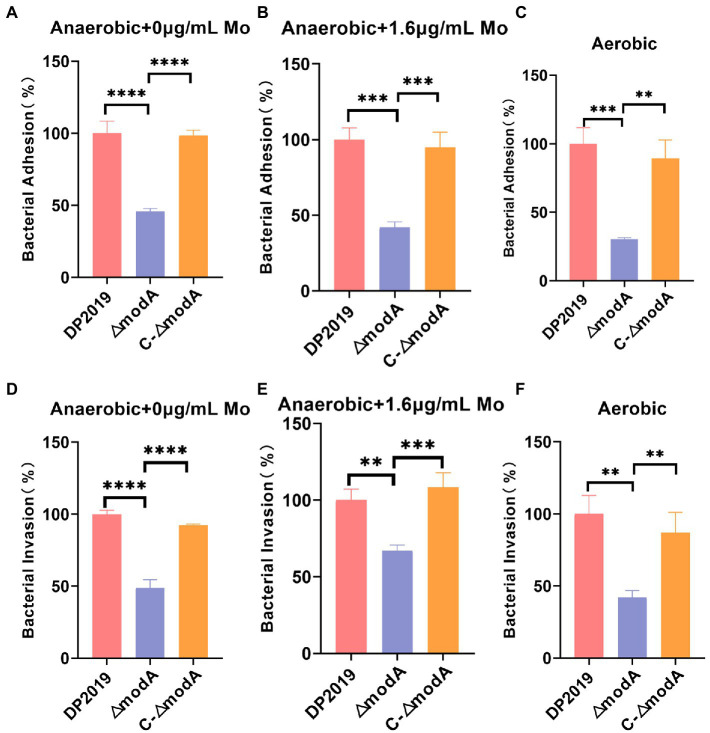
Effect of ModA on the virulence of *P. mirabilis in vitro*. **(A)** Adhesion to immortalized human bladder epithelium cells under anaerobic conditions was expressed as the percentage of adherent bacteria (CFU/mL) versus the total bacteria added (CFU/mL). Then DP2019 group was designated as 100% to calculate the relative rate of adhesion. **(B)** Adhesion to immortalized human bladder epithelium cells in 1.6 μg/mL molybdate under the anaerobic conditions to avoid the interference of anaerobic growth defects on the results. **(C)** Adhesion to immortalized human bladder epithelium cells under aerobic conditions. **(D)** Invasion of immortalized human bladder epithelium cells under anaerobic conditions, which was expressed as the percentage of viable bacteria (CFU/mL) that survived the gentamicin treatment versus the total bacteria added (CFU/mL). Then DP2019 group was designated as 100% to calculate the relative rate of invasion. **(E)** Invasion to immortalized human bladder epithelium cells in 1.6 μg/mL molybdate under the anaerobic condition to avoid the interference of anaerobic growth defects on the results. **(F)** Invasion to immortalize human bladder epithelium cells under aerobic conditions. Data are presented as the mean ± S.D. of three independent experiments, **p < 0.1, ***p < 0.001, ****p < 0.0001.

Further investigation was required to precisely describe the involvement of *modA* in the virulence of *P. mirabilis*. An *in vitro* cell model assay was performed using both wild-type strain DP2019, the *ΔmodA* mutant and the *C-ΔmodA*. Under anaerobic growth conditions, no matter whether the medium was suppled with high concentration molybdate or not, the cell adhesion ability and invasiveness of the *ΔmodA* mutant was significantly reduced relative to those of wild-type strain DP2019, which was not due to its anaerobic growth defects, but there was no significant difference between those of complemented strain *C-ΔmodA* and the wild-type strain (Figures 5A,B,D,E). Perinet et al. (2016) found that *modA* may be important for the global virulence network of *P. aeruginosa* in aerobic conditions. We decided to use the cell model under aerobic conditions. Our data show that under aerobic conditions, the ability of *modA* deletion mutant to adhere to and invade urinary tract epithelial cells is also reduced (Figures 5C,F). These assays highlight the fact that ModA may be important for the virulence of *P. mirabilis in vitro*, even in aerobic conditions, our findings preliminarily demonstrated that the loss of the ModA weakened the virulence of *P. mirabilis*.

### The *modA* mutant of *Proteus mirabilis* shows reduced virulence and tissue damage in a murine UTI model

**Figure 6:**
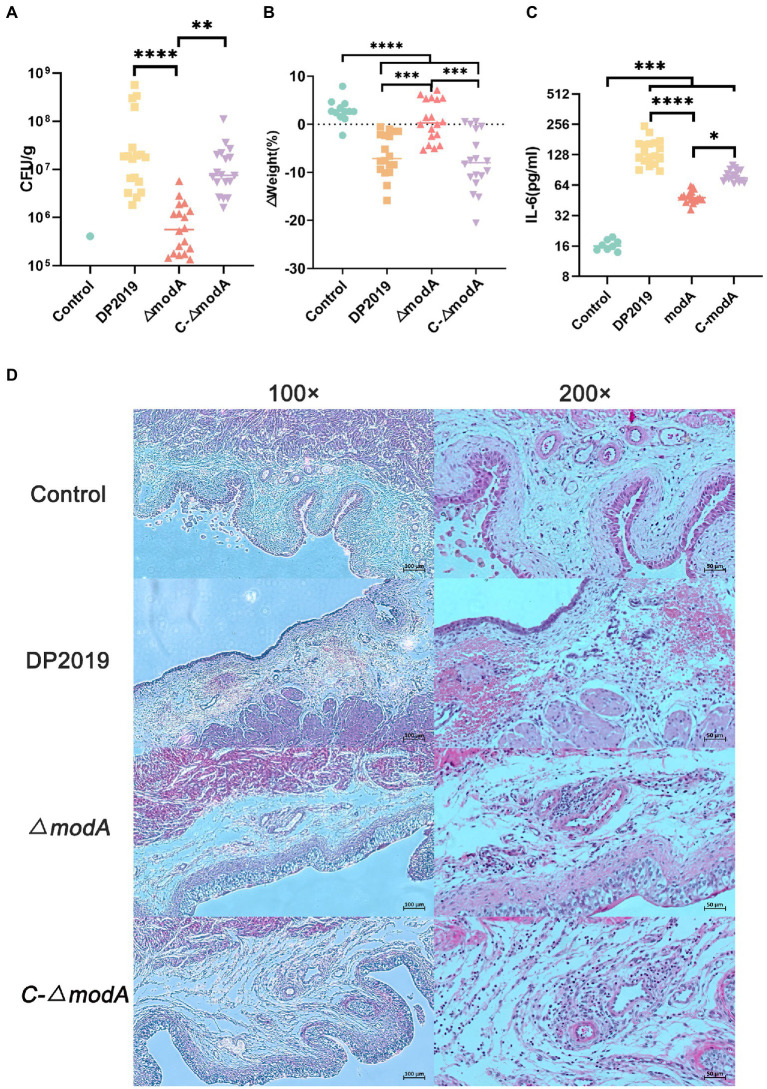
Mouse UTI infection model. **(A)** Levels of bacterial colonization in bladder tissue. Each square triangle represents CFU per gram of bladder tissue from an individual mouse. Horizontal lines represent the geometric means of the colony counts, *n* = 12 for the Control group, *n* = 18 for each UTI group. **p < 0.01, ****p < 0.0001. **(B)** Changes in mouse body weight before and after infection, it was represented as % from the original weight rather than absolute values, *n* = 12 for the Control group, *n* = 18 for each UTI group, ***p < 0.001, ****p < 0.0001. **(C)** IL-6 levels in the bladders of the mice, we used Elisa to detect the IL-6 in the bladder tissue in each group, *n* = 12 for the Control group, *n* = 18 for each UTI group. *p < 0.05, ***p < 0.001, ****p < 0.0001. **(D)** Hematoxylin–eosin (HE)-stained sections of the bladder. A histological examination of the H&E staining at 10× and 40× are displayed, respectively. The bladder tissue in the UTI group was significantly thicker than that in the control group, especially in DP2019 and *C-∆modA* groups. Under a 40× microscope, inflammatory cell infiltration, edema, and hemorrhage were shown in the lamina propria in the DP2019 group, inflammatory cell infiltration, and edema were also observed in the *C-∆modA* group, and the injury and inflammation were observed to be mild in the *∆modA* group, but no significant histological changes were found in the control groups.

We used a well-characterized UTI model to further study the contribution of ModA to the pathogenicity of *P. mirabilis* during UTI. Few bacteria were detected in the bladders of mice inoculated with sterile phosphate-buffered saline (PBS; control), whereas inoculation with 10^9^ cfu/mL *P. mirabilis* significantly increased bacterial numbers cultured from bladders of mice (UTI group, induced with strain DP2019, *ΔmodA, and* C-*ΔmodA*). In the UTI group, the bladders of mice inoculated with wild-type DP2019 or complemented strain C-*ΔmodA* showed high levels of bacterial colonization, whereas those inoculated with the *ΔmodA* strain showed significantly less colonization (Figure 6A). We also analyzed the weight changes in the mice before and after infection, and found that compared with the control group, the weight of the mice in the *ΔmodA* group did not change significantly, whereas that of the mice in the DP2019 and *C-ΔmodA* groups decreased, consistent with the observed infection symptoms in these mice, and indicating that the mice suffered systemic illness during infection (Figure 6B). Studies have shown that interleukin 6 (IL6) secretion was functionally significant during UTI and that IL6 levels increased as the severity of UTI increases (Ching et al., 2018). Therefore, we investigated the IL6 secretion in a murine model of UTI. The content of IL6 in the mice infected with wild-type strain DP2019 or *C-ΔmodA* was higher than that in the PBS-treated control group, whereas the IL6 levels in the mice infected with mutant *ΔmodA* were lower than those in the wild-type-infected and *C-ΔmodA*-infected mice (Figure 6C). We then observed pathological sections of the bladder tissues. It could be observed that the bladder mucosa in the control group was smooth and intact, without edema and inflammatory cell infiltration. However, the bladder mucosae in the UTI group showed more edema and inflammatory cell infiltration than those in the control group, but to varying degrees. Both symptoms were especially prominent in the DP2019-treated mouse tissues, which showed more neutrophil and lymphocyte infiltration, more interstitial edema with hemorrhage, and more reactive hyperplasia of the urinary epithelial cells than were observed in the tissues from mice treated with *ΔmodA* or *C-ΔmodA*. In the *C-ΔmodA*-infected mouse tissues, there was also obvious interstitial edema and infiltration of large numbers of inflammatory cells, whereas, in the *ΔmodA*-infected mouse tissues, edema and inflammatory cell infiltration were less severe (Figure 6D). In conclusion, these results indicated that the deletion of the *modA* gene reduced the virulence of *P. mirabilis*, thereby reducing the damage to the host during infection.

### ModA affects the expression of multiple genes in the pathway of pili assembly and bacterial adhesion

**Figure 7:**
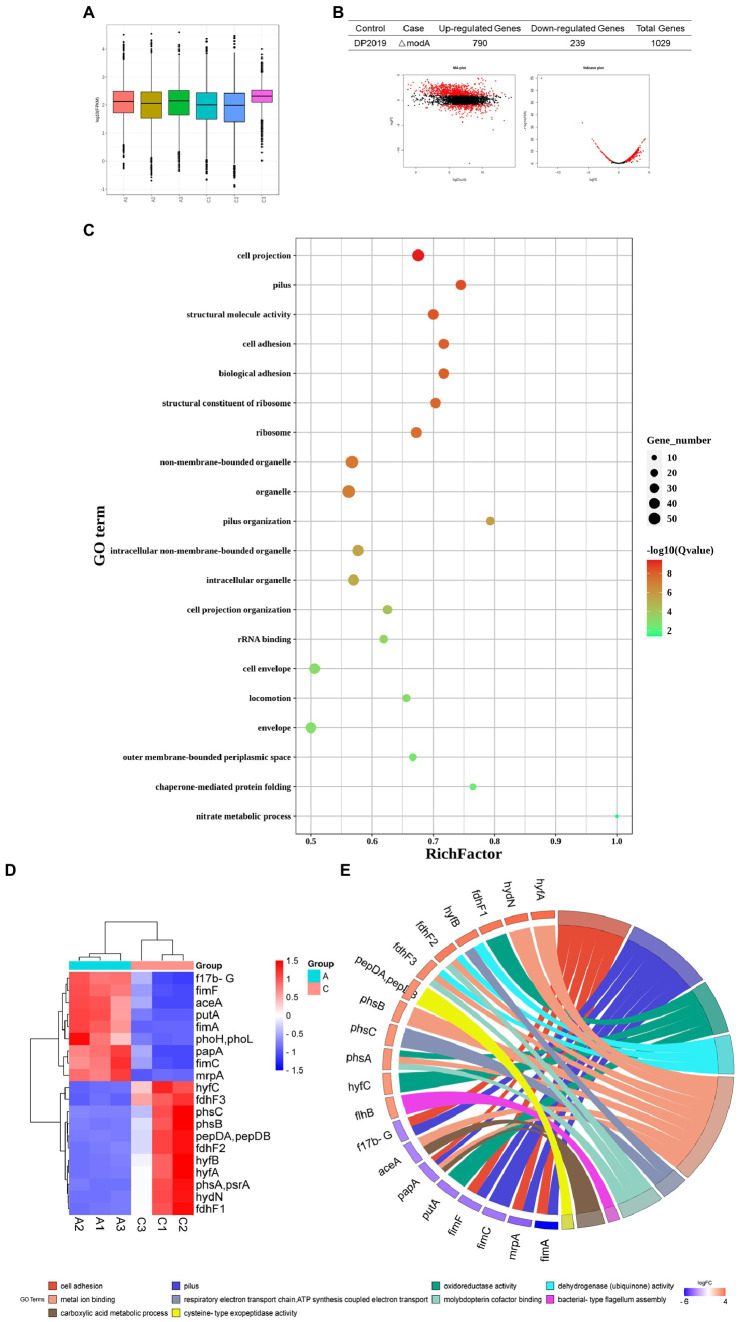
Functional analysis of DEGs. “A” represents the control group DP2019 and “C” represents the *ΔmodA* group. **(A)** FPKM box graph of different samples of DP2019 and *ΔmodA*. The abscissa shows the sample name, the ordinate shows log_10_ (FPKM). The box graph of each area corresponds to five statistics FIGURE 7 (Continued)(the maximum, upper quartile, median, lower quartile, and minimum values, from top to bottom). **(B)** Statistical table of differential gene expression, MA map, and volcano map. The number of upregulated differentially expressed genes met the conditions FDR < 0.05 and log_2_FC > 1, and the number of downregulated differentially expressed genes met the conditions FDR < 0.05 and log_2_FC < 1. **(C)** Differentially expressed gene GO enrichment scatterplot. The vertical axis shows the GO terms, which are sorted from smallest to largest according to *q* value. The horizontal axis shows-log10 (*q* value). The sizes of the points represent the numbers of DEGs in the GO terms. The colors of the points correspond to different *q* values. The larger the rich factor, the greater the enrichment. The range of the *q* value is [0, 1]; the closer to zero, the more significant the enrichment. **(D)** Differential gene expression clustering heat map. The 20 most significantly differentially expressed genes are shown. Blue represents low gene expression, and red represents high gene expression. **(E)** GO chord plot of the first 20 DEGs. The left side of the figure shows the target genes screened, and the right side shows the corresponding GO terms. Different colors represent different GO terms.

Transcriptome refers to the sum of all RNA that can be transcribed by a cell or tissue under a specific condition. It studies the gene function and gene structure from the overall level and comprehensively and rapidly obtains the identity and expression level of each gene (Dong and Chen, 2013). Therefore, the transcriptome is an important means to study the phenotype and function. In this study, to advance our understanding of the overall expression level affected by *modA* to *P. mirabilis* compared with its phenotype, we employed a transcriptomic approach to reveal the mechanism of gene regulation and explain genotype and phenotypic variation. The distributions of the fragments per kb of exons per million mapped fragments (FPKM) among the samples of DP2019 and *ΔmodA* were similar (Figure 7A). Our results showed that *modA* knockout (*ΔmodA* strain) affected the expression levels of 1,029 genes, 790 of which were upregulated and 239 of which were downregulated (Figure 7B). The distribution of the false discovery rate (FDR) and difference multiple fold change (FC) values of all the genes between the two groups of samples is shown in an MA plot and volcano map (Figure 7B). A gene ontology (GO) enrichment analysis showed that most differentially expressed genes (DEGs) were enriched in the GO terms “pilus” (GO: 0009289), “cell adhesion” (GO: 0007155), “biological adhesion” (GO: 0022610), “pilus organization” (GO: 0043711), “locomotion” (GO: 0040011), and “nitrate metabolic process” (GO: 0042126), consistent with the phenotypic test results described above. These findings demonstrate that the *modA* gene is crucial to bacterial nitrate metabolism, virulence, and pathogenicity (Figure 7C). We screened the 20 most significantly differentially expressed genes that were annotated in the GO database for expression profile analysis. As shown in Figure 7, compared with the DP2019 bacterial strain, the expression of the *f17b-G*, *fimF*, *aceA*, *putA*, *fimA*, *phoH*, *phoL*, *papA*, *fimC*, and *mrpA* genes was significantly downregulated in the *ΔmodA* strain, whereas the expression of the *hyfC*, *fdhF3*, *phsC*, *phsB*, *pepDA*, *pepDB*, *fdhF2*, *hyfB*, *hyfA*, *phsA*, *psrA*, *hydN*, and *fdhF1* genes was significantly upregulated (Figure 7D, constructed with bioinformatics). We drew the GO chords for these 20 DEGs, and the DEGs were enriched in GO terms such as “metal ion binding,” “pilus,” “cell adhesion,” “oxidoreductase activity,” “dehydrogenase activity,” “molybdopterin cofactor binding,” “reactive electric transport chain,” “ATP synthesis coupled with electric transport,” “carboxylic acid metadata process,” “bacterial type flagellum assembly,” etc. Among these, the downregulated DEGs were enriched in the GO terms “pilus,” “cell adhesion” and “cellular acid metadata process,” whereas the upregulated DEGs were enriched in the GO terms “dehydrogenase activity,” “molybdopterin cofactor binding,” “reactive electron transport chain,” “ATP synthesis coupled with electric transport,” and “bacterial type flexible assembly.” The GO terms “metal ion binding,” and “oxidoreductase activity,” contained both up-and downregulated DEGs (Figure 7E, constructed with bioinformatics). These data suggested that a defective *modA* gene affected the expression of multiple genes in the pathway of pili assembly and bacterial adhesion, consistent with the results of our *in vitro* cell model and *in vivo* animal model assays.

### ModA affects the expression of multiple genes in the flagellum pathways

**Figure 8:**
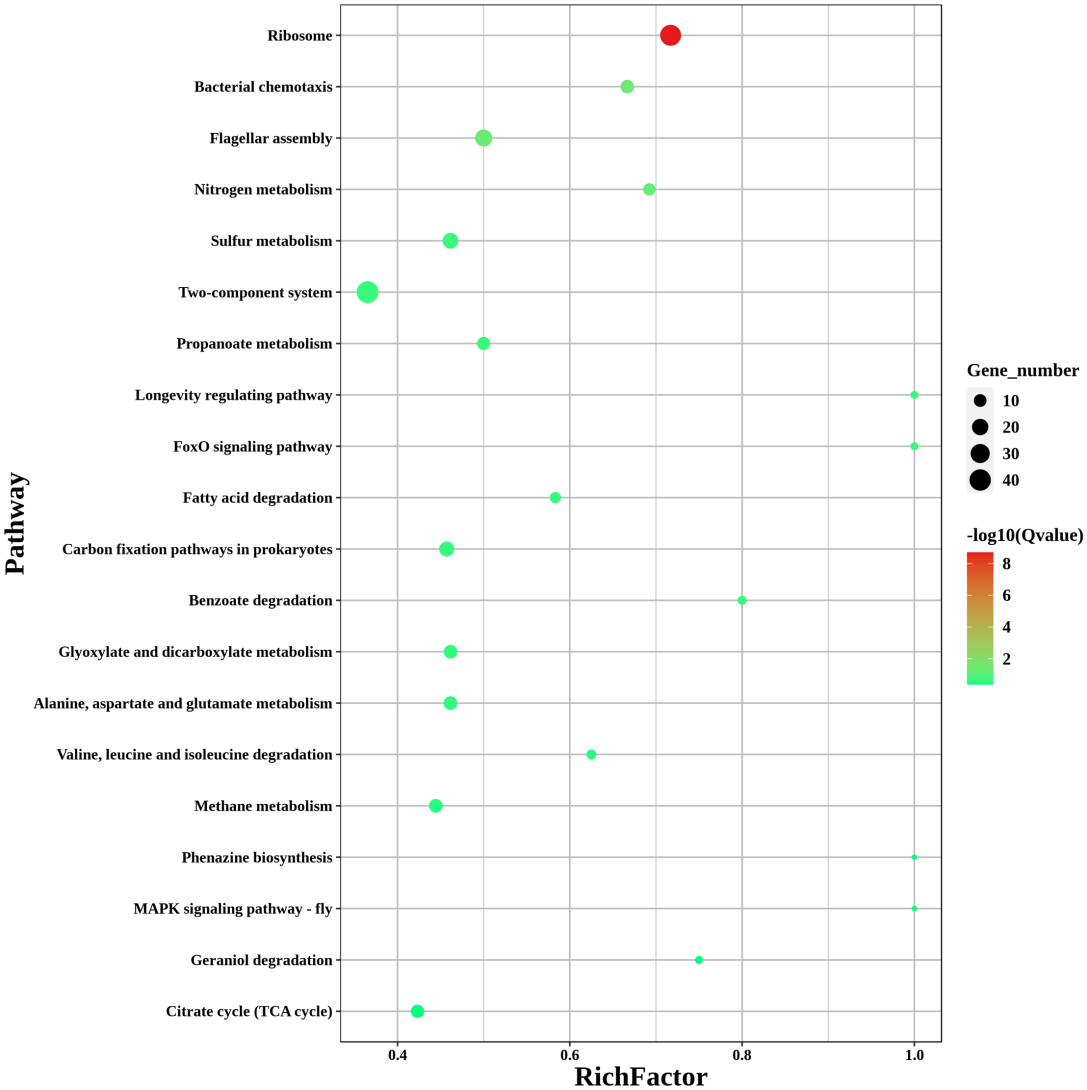
KEGG pathways of DEGs. KEGG enrichment scatterplot. The vertical axis shows the path name, sorted from smallest to largest *q* value; the horizontal axis shows-log10 (*q* value); the sizes of the points indicate the numbers of DEGs in the pathways; the colors of the points correspond to different *q* value values. The greater the rich factor, the greater the enrichment. The range of the *q* value is [0, 1]; the closer to zero, the more significant the enrichment.

To investigate the pathways significantly associated with *modA*, a KEGG pathway enrichment analysis was performed. The results showed that the DEGs in *ΔmodA* were significantly enriched in the “flagellar assembly” and “nitrogen metabolism” pathways (Figure 8). We also observed that a panel of flagella-assembly (KO02040) genes was upregulated in the *ΔmodA* knockout strain of *P. mirabilis* (Supplementary Figure S1) (Aizawa, 2001; Aldridge and Hughes, 2002; Liu and Ochman, 2007; Morimoto and Minamino, 2014; Osterman et al., 2015; Khan et al., 2020; Takekawa et al., 2020). These data indicated that loss of ModA resulted in alterations in the expression of genes linked to the flagella assembly pathway.

### Validation of DEGs with RT–qPCR

**Figure 9:**
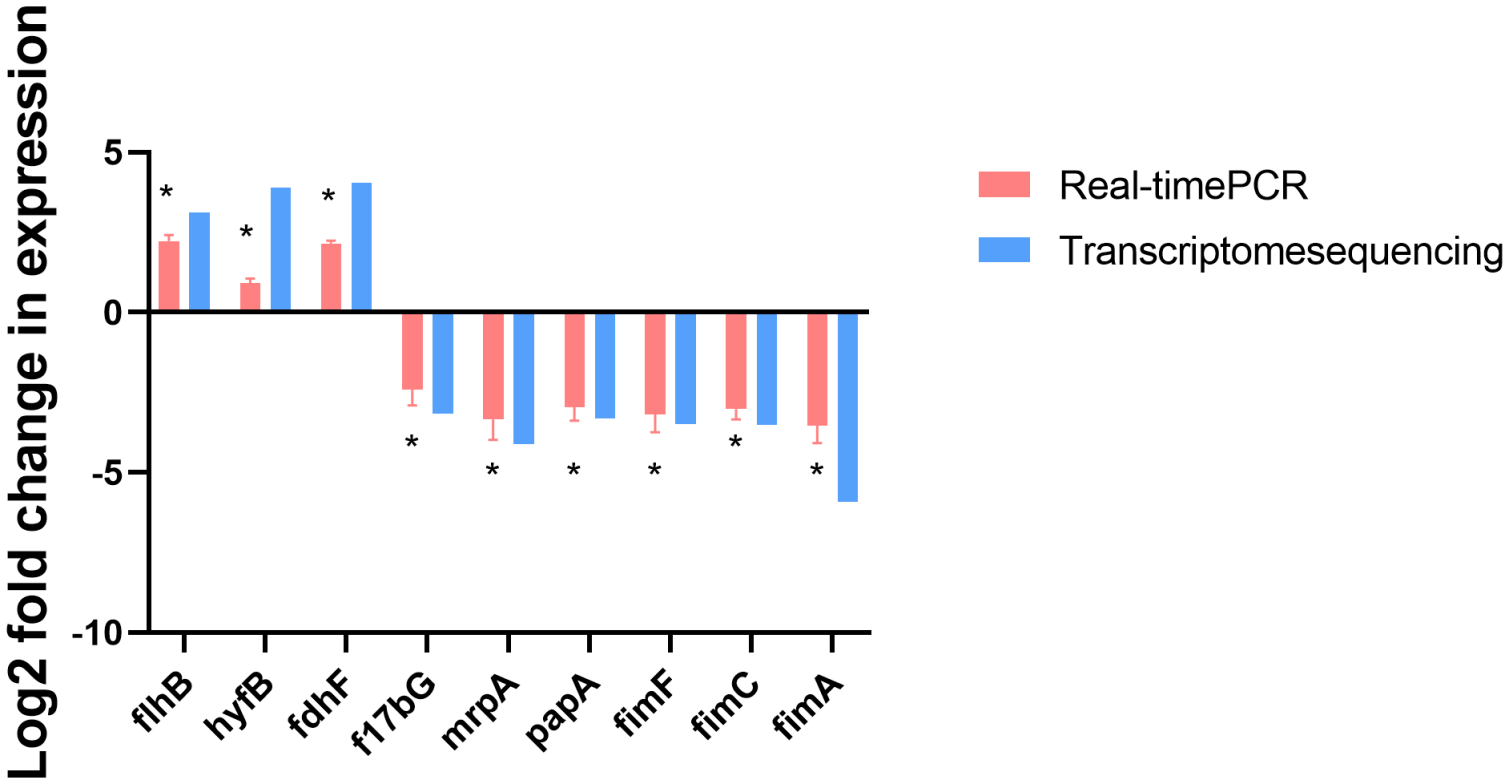
RT-qPCR confirmation of the three upregulated and six downregulated genes in *ΔmodA* relative to DP2019. Values are the means of at least three independent assays. Error bars indicate standard deviations, *p < 0.05.

The gene transcription profiles of the genes upregulated and downregulated in the *ΔmodA* and DP2019 strains were determined with a differential analysis, followed by RT–qPCR to verify the three most significantly upregulated and six most significantly downregulated genes. RT–qPCR showed that the expression of nine genes was consistent with the results of the transcriptome analysis. Both RT-qPCR and transcriptomics showed that the expression of the *flhB*, *hyfB*, and *fdhF* genes was significantly upregulated, whereas the expression of the *f17bG*, *mrpA, papA, fimF, fimC*, and *fimA* genes was significantly downregulated with both methods. Among these genes, the *flhB* gene acted in the flagellar assembly pathway, and the *mrpA, papA, fimF, fimC*, and *fimA* genes acted in bacterial pilus assembly and virulence (Figure 9).

## Discussion

Molybdenum, an essential trace element for many organisms, is transported by the high-affinity transporter ModA, encoded by the *modA* gene, in many kinds of bacteria. Recent research has examined the high affinity of ModA for molybdate in different species (Hoffmann et al., 2016; Perinet et al., 2016; Xia et al., 2018; Ge et al., 2020; Rahman et al., 2021; Wang et al., 2021). The molybdate transported into bacteria is incorporated into the molybdopterin molecule to produce MoCo. Moco cofactors can be further modified for use by molybdenum-containing enzymes. The NarGHI complex is a typical molybdenate-containing enzyme that plays a role in the initial stage of nitrate reduction to nitrite (Bertero et al., 2003; Kraft et al., 2011; Pederick et al., 2014). Nitrate serves as a terminal electron acceptor under anaerobic conditions in *Ps. aeruginosa*. Thus this molybdenate-containing enzyme participates in the anaerobic growth of bacteria (Van Alst et al., 2009). Therefore, the ModA protein plays a pivotal role in nitrate reduction and energy production in bacteria under anaerobic conditions (Kraft et al., 2011; Pederick et al., 2014; Maunders et al., 2022). Several studies have shown that under anaerobic conditions, the nitrate reduction rate of a *modA*-mutant strain was reduced and its nitrate reductase activity was also reduced. When the growth medium was supplemented with about 1 μM of sodium molybdate, the nitrate reductase activity of the mutant was restored to the wild-type levels. Molybdate supplementation had no effect on wild-type growth (Perinet et al., 2016). The growth of *modA* mutants in LB medium supplemented with KNO_3_ was impaired, but the growth rates of these strains were the same under aerobic conditions (Pederick et al., 2014; Perinet et al., 2016; Wang et al., 2021). Based on these findings, we hypothesized that the *modA* gene product was involved in the high-affinity transport of molybdate. Nitrate reductase activity might also be reduced in the absence of *modA*, and might only occur in the presence of traces of molybdate in the medium. As a consequence, *P. mirabilis* anaerobic growth might be inhibited. As in previous studies, we used ICP-MS to confirm the role of ModA in the uptake of molybdate by *P. mirabilis*. When molybdate is transported into *P. mirabilis*, it participates in the synthesis of molybdoenzymes. Nitrate reductase, a molybdoenzyme, plays a role in the initial stage of anaerobic respiration. We assayed the nitrate reductase activity to find the nitrate reductase activity of *P. mirabilis ΔmodA* decreases significantly under anaerobic conditions in the presence of trace molybdate. The decreased enzyme activity may indicate that reduced molybdate availability results in poorer nitrate reductase activity based on the fact that restoration of nitrate reductase activity is regained when molybdate levels are increased for the mutant strain. It was previously demonstrated that independent of ModA, there is a low-affinity system called the sulfate transport system (cysTWA product) and a non-specific anion transport system, which requires a higher concentration of molybdate (Gisin et al., 2010; Tirado-Lee et al., 2011; Hille et al., 2014). Therefore, we infer that there is a low-affinity system that transports molybdate to participate in the synthesis of molyboenzyme at high molybdate concentration. A growth assay showed that the deletion of *modA* inhibited the anaerobic growth of the mutant in the presence of nitrate in the presence of traces of molybdate. Therefore, ModA participated in the synthesis of molybdoenzyme by transporting molybdate, and the deletion of *modA* reduced the nitrate reductase activity and anaerobic growth of *P. mirabilis* at low molybdate concentrations. In UTIs, mixed infections of multiple bacteria increase the demand on the nitrate reductase system, and patients with UTIs caused by *P. mirabilis* are also susceptible to biofilm formation, leading to the formation of an anaerobic environment (Nzakizwanayo et al., 2019). Hence, ModA was involved in the anaerobic growth and nitrate utilization of *P. mirabilis* in the presence of nitrate, and its influence on *P. mirabilis* virulence and UTI warrants further study.

The association between ModA and the virulence and pathogenicity of *P. mirabilis* has been confirmed. Studies have shown that the deletion of *modA* significantly improved the survival rate of mice with acute pneumonia by *Ps. aeruginosa* (Wang et al., 2021). In a rat model of cystic fibrosis-associated infection, *modA* was important for bacterial virulence. ModA enhances the resistance of the bacteria to the amoeba *Dictyostelium discoideum* to predation under aerobic conditions (Perinet et al., 2016). In a lettuce model and a burned mouse model of acute toxicity, molybdate was crucial to the toxicity and biofilm maturation of the infecting bacteria (Filiatrault et al., 2006, 2013). Genes related to molybdenum utilization and transportation (*moe* and *moa*) are also important for bacterial pathogenicity (Williams et al., 2014; Levillain et al., 2017). It has been reported that a transposon insertion mutation of *modA* weakened the proliferation and virulence of *M. tuberculosis* in the lungs of mice (Camacho et al., 1999). Tungstate, a competitive inhibitor of molybdenum, can eliminate the adaptive advantages of MoCo, selectively inhibiting the expansion of Enterobacteriaceae, and thus improving intestinal inflammation (Zhu et al., 2018). It is noteworthy that with the mutation of multiple genes (including *modA*) related to molybdenum, a carcinogenic *Helicobacter pylori* strain seemed to lose the ability to import molybdate or synthesize molybdopterin (Kawai et al., 2011). This emphasizes the particular importance of further studying the relationship between the *modA* gene and bacterial virulence, pathogenicity, host health, and disease status. Therefore, we inferred that ModA had a corresponding regulatory effect on the virulence of *P. mirabilis*. *P. mirabilis* uses various sets of virulence factors to access and colonizes the host urinary tract to regulate pathogenesis, including fimbriae and other adhesins, and biofilm formation, which are induced during swarming compared to broth culture or older bacteria in the interior of a swarm colony (Schaffer and Pearson, 2015). Our research into the virulence of *P. mirabilis* included the analysis of its swarming and swimming motility, its ability to form biofilm, and its adhesion to and invasion of urothelial cells.

*Proteus mirabilis* is best known for its swarming motility on solid surfaces and swimming motility on liquid media, which may contribute to the prevalence of *P. mirabilis* in the UTIs associated with urinary catheters (Warren et al., 1982; Jones et al., 2004; Armbruster et al., 2017). In a past study, we isolated two strains of *P. mirabilis*, G121 and G137, with abnormal migration ability from patients in the early stage of clinical UTIs, and used transcriptomics to show that the expression of the *modA* gene was significantly upregulated in them (Peng et al., 2020). Many researchers believe that flagella are an extremely important factor in the swarming motility and swimming motility of *P. mirabilis* (Alavi et al., 2001; Rather, 2005). Our swarming motility and swimming motility assay, combined with the previous study by Peng et al. (2020), emphasized the fact that even under aerobic conditions, *modA* might be important for the swarming and swimming motility of *P. mirabilis*. A functional classification and enrichment analysis showed that the deletion of *modA* upregulated the expression of many genes, which were significantly enriched in the KEGG “flagella assembly” pathway, suggesting that the loss of ModA may result in increased expression of genes associated with flagella and motility. However, previously described reports in which non-motile *P. mirabilis* was fully virulent suggest that swarming may not be an important contributor to UTI virulence (Schaffer and Pearson, 2015). RT–qPCR confirmed that the *flhB* gene, which acts in the flagellar assembly pathway, was significantly upregulated in *ΔmodA*. Combined with our transcriptomic results, we infer that the effect of ModA on swarming motility and swimming motility may be related to bacterial flagellar assembly.

In clinical scenarios in which catheters are used, catheter-related UTI has become the commonest infection in many medical care systems. *Proteus mirabilis* is a particularly problematic pathogen in this context. One of its main characteristics and major complications is its formation of large numbers of crystalline biofilms (Stickler, 2008; Holling et al., 2014a,b). Perinet et al. (2016) observed the formation of a weak biofilm by a *Ps. aeruginosa modA* mutant, but its reduced biofilm formation was irrelevant to its anaerobic growth defects. It has also been demonstrated that molybdate was crucial for the maturation of biofilm (Filiatrault et al., 2013). The formation of a weak biofilm by our *P. mirabilis modA* deletion mutant indicated that ModA affected biofilm formation under anaerobic, which was not due to anaerobic growth defects. Our transcriptomic analysis combined with RT–qPCR showed that *mrpA* was significantly downregulated in the *ΔmodA* mutant. Since Pearson reported that MR/P fimbriae contributed to biofilm formation *in vitro* (Pearson, 2019), we speculated that the reduced biofilm formation ability of the *ΔmodA* mutant was related to the downregulated expression of the *mrpA* gene.

In an *in vitro* cell model, the loss of ModA significantly reduced the adhesion and invasiveness of *P. mirabilis* under anaerobic conditions. The decrease of adhesion and invasion of *P. mirabilis* to urothelial cells *in vitro* was not due to its anaerobic growth defects. Even under aerobic conditions, ModA may be important for the adhesion and invasion of *P. mirabilis.* To better understand the effect of ModA on the UTIs caused by *P. mirabilis*, we developed a murine UTI model. We found that the deletion of *modA* significantly reduced the ability of *P. mirabilis* to colonize bladder tissue. It was found that the level of IL6 correlates positively with the severity of UTIs (Wood et al., 2011; Ching et al., 2018). Besides, the *in vivo* levels of cytokine IL6 were significantly reduced in the bladder tissues infected with the *ΔmodA* mutant relative to that in DP2019-or *C-ΔmodA*-infected bladder tissues. Urinary tract infections caused by *P. mirabilis* can stimulate the inflammatory reaction of the bladder tissue section. Histological analysis also demonstrated that the *ΔmodA* mutant caused less inflammatory damage to the bladder than the other two strains. Since the environment is likely to be microaerobic, the diminished virulence may be a direct result of loss of ModA, or a result of reduced final titer in *modA* mutant under anaerobic, or the result of the joint action of the two factors. In a word, ModA plays an important role in *P. mirabilis*-induced UTIs. A comparative transcriptomic analysis and RT-qPCR assay show that *f17b-G*, *mrpA*, *fimA*, *fimF*, *fimC*, and *papA*, which are related to pilus formation, cell adhesion, and virulence, were significantly downregulated. MR/P fimbriae are considered to be major contributors to UTIs (Schaffer and Pearson, 2015). Taken together, we speculate that the role of ModA in *P. mirabilis* virulence is critical, and in its absence expression of certain genes required for the synthesis of pili/adherence factors may be decreased.

In this study, to extend our understanding of the role of ModA in bacterial transport, growth, metabolism, and pathogenicity, transcriptomic analysis was used to examine the corresponding mechanisms. Because our prior researchers found the decline of migration ability of clinical strains with high expression of *modA* in transcriptomics carried out under aerobic conditions, the above phenotypic experiments were also tested under aerobic conditions, and transcriptomics was also explored under the same conditions. We have provided evidence that ModA bound molybdate with high affinity and transported it to the site of molybdoenzyme synthesis and consequently affected the anaerobic growth of *P. mirabilis*. That is, the loss of ModA reduced its anaerobic growth at low molybdate concentration. However, it enhanced the swarming and swimming motility of *P. mirabilis*. The anaerobic growth of *P. mirabilis* is closely related to its biofilm formation during UTI (Yu et al., 2021). This prompted us to find that the loss of ModA reduced its biofilm formation capacity, which leads to bacterial susceptibility to the host immune system and the elimination of antibiotics during UTI. Besides, the reduced ability to adhere to and invade epithelial cells in *modA* mutant weakened the inflammatory damage it caused to the host during UTI. Taken together, we have used a series of *in vitro* virulence assays and an *in vivo* UTI infection model, combined with a transcriptomic analysis and RT-qPCR, to provide comprehensive evidence of the important role of the ModA protein in the pathogenicity of *P. mirabilis*. However, since the deletion of *modA* contributes to multiple genes upregulated in flagellum assembly pathway and downregulated in fimbriae assembly pathway, we hypothesize that there is an indirect effect of *modA* on UTI virulence, which provides new insights into how *P. mirabilis* causes UTIs through ModA. These findings illustrated the different physiological and pathogenicity-related functions of ModA in *P. mirabilis*. Our data provided a theoretical basis for understanding the pathogenic mechanisms of *P. mirabilis*-induced UTIs. A comprehensive analysis of the role of ModA in the UTIs caused by *P. mirabilis* should offer new strategies for clinical therapies.

## Materials and methods

### Bacterial strains and culture conditions

*Proteus mirabilis* strain DP2019 (accession number: CP110673) was clinically isolated from a patient with a UTI. The *modA*-deletion mutant was constructed and identified with homologous recombination technology using strain DP2019. *E. coli* β2155 and plasmid pCVD442 were used for making the isogenic deletion mutant of *modA*. The upstream A and downstream B homologous recombination arms of the *modA* gene were amplified from the genome of DP2019 with DNA polymerase. The A fragment (0.79 kb) was amplified with primers modA-AF (5′-CAGTTGCTCTATCAACCAGGAGTTC-3′) and modA-AR (5′- CGCACCAGCCAGTAACTTACCTG-3′). The B fragment (0.8 kb) was amplified with primers modA-BF (5′-TACGGCTTTAGCCCACTTTAGGAATTATAC-3′) and modA-BR (5′- CGTGATACTTTCTGTTGGTAATTGAGTATTCAC-3′). The *Kn* resistance gene (kanamycin resistance gene, 0.82 kb) was amplified from pET28a plasmid with primers modA-KnF (5′- CAGGTAAGTTACTGGCTGGTGCGTCATGAACAATAAAACTGTCTGC-3′) and modA-KnR (5′- GTATAATTCCTAAAGTGGGCTAAAGCCGTATTAGAAAAACTCATCGAGCATC-3′). Connect the upstream and downstream homologous recombinant arm of *modA* gene with *Kn* resistance gene through fusion PCR technology to obtain the complete target fragment *ΔmodA:: Kn* (2.54 kb), with an internal deletion of the *modA* gene containing SalI and EcoRV restriction sites. The *ΔmodA:: Kn* fragment was inserted into pCVD442 between SalI and EcoRV restriction sites, and the resulting recombinant plasmid was designated. It was transferred into *E. coli* β2155 by electrotransformation and the recipient strain of *P. mirabilis* carried out the conjugation experiment. The kanamycin-resistant *P. mirabilis* clone was screened on the kanamycin plate. Its genome was integrated with a target plasmid, called DP2019/pCVD442-ΔmodA::Kn. DP2019/pCVD442-ΔmodA::Kn clone liquid was inoculated with LB plate containing kanamycin and 10% sucrose and then cultured until monoclone was formed. A clone with *modA* gene replaced by the *Kn* resistance gene was obtained through PCR screening and designated *ΔmodA*.

Plasmid pBR322 carrying an ampicillin resistance gene was used for the construction of the *modA*-complemented strain. *modA gene* fragment (0.80 kb) was amplified from the original strain of DP2019 with primers modA-F (5′-CAATGCGCTCATCGTCATCCTC-3′) and modA-R (5′-CACCTGTCCTACGAGTTGCATG-3′), containing SalI and EcoRV restriction sites and was cloned into the corresponding site of pBR322 to obtain the complementary plasmid pBR322-modA. pBR322-modA complement plasmid was transformed into *ΔmodA* strain by electrotransformation, and positive clones were screened on an ampicillin plate. The purified pBR322-modA plasmid was added to the receptive cells, transferred to the BioRad, and transformed by an electric shock at 2,500 V (BioRad MicroPulser). The transformed bacterial solution was immediately suspended with fresh LB and incubated at 37°C and 220 rpm for 2 h. Take the resuscitated transformation medium and lay it on an LB plate (including Amp 100 μg/mL), cultured at 37°C until monoclone formation. Combined with PCR and sequencing technology, the retroactive plasmid was confirmed to be transferred into the target strain, and one of the clones was designated C-*ΔmodA*.

For routine culture, *P. mirabilis* was grown aerobically at 37°C in LB broth (Solarbio). When needed, the culture medium was supplemented with 1% (w/v) bacto agar, kanamycin (Kn, 20 μg/mL; Sigma-Aldrich), or ampicillin (Amp, 100 μg/mL for *C-ΔmodA*; Sigma-Aldrich).

### Inductively coupled plasma mass spectrometry (ICP-MS)

For ICP-MS, each bacterial sample was inoculated to the blood agar plate medium, and culture at 37°C overnight. Gently weighed and placed into microwave, add 5 ~ 10 mL of nitric acid, cover it, and place it for 1 h or overnight, screw the tank cover tightly, and digest it according to the standard operation steps of the microwave digestion instrument. High-pressure sealed jar for digestion on a temperature-controlled electric heating plate, heat it at 100°C for 30 min or ultrasonic degassing for 2–5 min, add water to a constant volume of 25 or 50 mL, and mix well for standby. A blank sample was prepared in parallel. According to the standard operating procedure of the ICP-MS apparatus, the instrument was tuned to meet the measurement requirements, the measurement method was edited, and the internal standard element, 103Rh/115In, appropriate for molybdenum (Mo) was selected. The m/z of Mo and 103Rh/115In was 95. The mixed standard solution was injected into the apparatus, and the signal response values for Mo and 103Rh/115In were measured. A standard curve was constructed, with the concentration of Mo as the abscissa and the ratio of the response signal value for Mo to that of the selected internal standard element as the ordinate. Inject the A blank solution and sample solution were injected into the apparatus, and the signal response values for Mo and 103Rh/115In were measured. The concentration of Mo in the digestion solution was determined from the standard curve.

### Nitrate reductase activity assay

Bacteria were grown under anaerobic conditions in a medium supplemented with potassium nitrate (KNO_3_, 20 mM; Solarbio) for 12 h. The cells (1 mL) were harvested by centrifugation at 2,500 × *g*, washed, and resuspended in 50 mM phosphate buffer (pH 7.2). Nitrate reductase activity was detected with the Nitrate Reductase (NR) Activity Detection Kit (Griess Colorimetric Method, Solarbio).

### Growth assay

For the growth experiments, *P. mirabilis* was grown aerobically with vigorous shaking (150 rpm) in 5 mL of LB broth at a dilution of 1:100. For anaerobic growth, the strain to be cultured was placed in an anaerobic tank (Oxoid), which was immediately wrapped in an anaerobic gas bag. The bag was sealed immediately, and the tank was transported to an incubator. A change in the anaerobic indicator from pink to white indicated an anaerobic status. The media for aerobic and anaerobic growth were supplemented with potassium nitrate (KNO_3_, 15 mM; Bolinda) and different volumes of 100 μg/mL molybdenum standard solution (Macklin) was added for anaerobic growth as required (Perinet et al., 2016). Growth was monitored with a microplate reader as the optical density at a wavelength of 600 nm (OD_600_). The growth experiments were repeated three times.

### Swarming motility and swimming motility assay

Swarming was assayed on agar plates containing 1.5% (w/v) agar and 5% (v/v) defibrinated sheep blood. An overnight culture was inoculated with a preculture in a volumetric ratio of 10:1 and incubated overnight with shaking at 37°C in optimal LB broth until the culture reached an OD_600_ of ~1.0. The culture density of each sample was adjusted to OD_600_ = 1.0. An aliquot (10 μL) of the overnight culture was carefully pipetted onto the center of the agar surface without puncturing the agar with the pipette tip. The plate was then dried for 20 min before it was inverted and placed in a 37°C incubator overnight. The swarm radius from the edge of the inoculation perimeter was observed, measured with a ruler, and photographed every 2 h.

Swimming was assayed on agar plates containing 0.25% (w/v) agar, 1% (w/v) tryptone, and 0.5% (w/v) NaCl. Adjust the overnight culture density to an OD_600_ of ~1.0. An inoculating needle was sterilized in a flame, then was allowed to cool. The standardized culture was dipped by the sterile needle. The needle was inserted vertically into the swimming motility agar without being pushed to the bottom of the petri dish. The needle was retracted vertically. The plate was then incubated in a humid 30°C incubator without being inverted. A diffuse colony spreading outward from the inoculation point was observed, measured with a ruler, and photographed every 2 h.

### Biofilm formation assay

A 96-well-plate rapid biofilm formation assay was performed. Two staining methods were used to dye the biofilms. For the Crystal Violet staining method, the strains were grown overnight in LB medium supplemented, when needed, with Kn or Amp. Measure the OD_600_ value of each group, and adjust the OD value of each group to be consistent (about 0.8–1.0). Each well of 96-well-plate was inoculate with standardized bacterial solution at a ratio of 1:100. When needed, 1.6 μg/mL molybdate was added to eliminate interference from anaerobic growth defects in the mutant. After static incubation under anaerobic conditions, the biofilms were stained with crystal violet every 2 h. Biofilm formation was quantified by dissolving the stain in 2 × 200 μL of 95% (v/v) ethanol. Biofilm formation was monitored with a microplate reader as the optical density at a wavelength of 570 nm (OD_570_). The experiment was performed three times.

The strain was incubated overnight in LB medium supplemented with Kn or Amp as needed. The bacteria were transferred to a 96-well plate for static culture under anaerobic conditions, and the biofilm was stained after 6, 12, or 24 h. The two staining components provided with the LIVE/DEAD^®^ BacLight^™^ Bacterial Viability Kit were applied as a 1:1 mixture to the bacterial suspension and mixed thoroughly. The samples were incubated at room temperature in the dark for 15 min. Discard The liquid was discarded and the samples observe under a fluorescence microscope.

### Cell culture

Human uroepithelial cell line (SV-HUC-1) was purchased from Guangzhou Yeshan Biotechnology Co., Ltd., and grown in DMEM: F12 (gibco, China) medium supplemented with 10% heat-inactivated fetal bovine serum (FBS, gibco, China), and then placed in a cell incubator.

### Cell adhesion and invasion assay

The cell adhesion assay was performed as described by Peng et al. (2016). Briefly, immortalized human bladder epithelium cells were grown in DMEM:: F12 (gibco, China) medium supplemented with 10% heat-inactivated fetal bovine serum, and penicillin G (50 μg/mL) or streptomycin (100 μg/mL) if needed, at 37°C under 5% CO_2_. Approximately 1 × 10^5^ cells/well were cultured in a 24-well plate with 1 × 10^7^ colony-forming units (CFU)/well of bacteria for 3 h. After incubation, the monolayers were washed four times with PBS and then suspended in 0.5% Triton X-100 for 8 min. The CFU of bacteria was counted on normal (non-swarming) agar plates. Adhesion was expressed as the percentage of adherent bacteria versus the total bacteria added. To calculate the relative adhesion rate, the DP2019 group was designated as 100%, and the relative adhesion rate was expressed as the ratio of the adhesion rate of the *ΔmodA* or *C-ΔmodA* group to that of the DP2019 group.

In the invasion experiment, after the cells and bacteria were incubated for 3 h, gentamicin with the final concentration of 100 μg/mL was added to each well for incubation to kill the extracellular bacteria, PBS was washed for 3 times, and the cells were lysed in the same way as adhesion, and the gradient dilution coating plate count of samples from each well was taken as the number of bacteria invading the cells. The invasion was expressed as the percentage of viable bacteria that survived the gentamicin treatment versus the total bacteria added. To calculate the relative invasion rate, the DP2019 group was designated as 100%, and the relative invasion rate was expressed as the ratio of the invasion rate of the *ΔmodA* or *C-ΔmodA* group to that of the DP2019 group.

### *In vivo* mouse assay

A total of 72 female C57BL/6 mice, aged 6–8 weeks (Beijing Vital River Laboratory Animal Technology Co., Ltd), were used in this study. To examine the effects of the *modA* gene on the pathogenicity of bacteria causing UTI, the mice were randomized to four groups transurethrally inoculated with either sterile PBS (control group), DP2019 strain, *ΔmodA* strain, or *C-ΔmodA* strain. The OD_600_ of each overnight culture was adjusted to ~1.000 for consistency, equivalent to a culture density of about 2–3 × 10^9^ cfu/mL. The adjusted cultures were used as the inoculant. Each mouse was weighed and anesthetized, and their bladders were gently pressed to discharge excess urine. A 24G needle soaked in 50% glycerin was inserted into the urethra, and gentle pressure was applied to ensure that it entered the urethra smoothly. If there was resistance at the pubic symphysis, make the needle flat so that the hose of the needle could slide into the bladder, and sterile forceps were used to clamp the remaining needle to fix it in place. A 50 μL microinjector containing 50 μL of mixed inoculum was inserted into the 24G indwelling needle, and the inoculum was slowly and evenly injected to ensure that it did not leak from the urethral orifice or the indwelling needle. The infection process was repeated after 24 h, and this point was considered the starting point of infection. The mice were weighed after 72 h and humanely euthanized. The bladders were approximately aligned along six points between the bladder neck and the median longitudinal axis of the top bladder wall. Half of the bladder that was cut was used to make pathological sections and the other bladder was weighed and homogenized in 500 μL of PBS. The tissue homogenate was continuously diluted to form a gradient and used to uniformly coat LB agar plates (containing 3.5% agar) for overnight culture. The number of colonies was then counted, and the count was converted to the tissue bacterial load (CFU/g). The change in the weight (△Weight%) was represented as % from the original weight.

### Hematoxylin and eosin (H&E) staining

A part of the bladder tissue was immediately fixed in a 4% paraformaldehyde solution (BL539A, Biosharp, China) for the paraffin section. After the fixed bladder tissues were washed, dehydrated, transparent, and embedded in wax, they were cut into 3 μm slices. Then put them into the water at about 40°C and make them fully flattened. After ironing, marked clean anti-slip slides successively, affixed the slices, and placed in a 60°C thermostatic oven to bake overnight. Subsequently, after the slices were dewaxed and rehydrated, stained with hematoxylin solution (G1003, Servicebio, China) for 5 min (min), and rinsed the excess dye solution on the slides with tap water. Eosin solution (G1003, Servicebio, China) was used to stain the slices for about 5 min. After the slices were dehydrated and became transparent. Finally seal with neutral gum (10004160, SCRC, China). Observe with microscope inspection, and image acquisition analysis (NIKON ECLIPSE E100, Nikon, Japan).

### Quantification of IL-6

The homogenate of bladder tissue was centrifuged at 4,000 rpm for 30 min, and the supernatant was extracted for detection. An ELISA was performed according to the manufacturer’s instructions (Mouse Interleukin 6 [IL-6] ELISA Kit, Cusabio). Reagents, samples, and standard solutions were prepared as directed and allowed to come to room temperature before use. A series of known IL6 concentrations (1.56–100 pg/mL) was run in parallel. Data from technical replicate wells were averaged to obtain the mean value per sample. The plates were manually washed and aspirated between steps to lower the background noise. Once the plates had been incubated appropriately, the OD_450_ was read with the Bio-Rad xMark^™^ Microplate Absorbance Spectrophotometer.

### RNA isolation, library construction, and sequencing

TRIzol Reagent (Takara) was used to extract total RNA according to the manufacturer’s instructions. LB broth (5 mL) was inoculated with strains DP2019, *ΔmodA*, or *C-ΔmodA* in a ratio of 100:1, and the strains were cultured at 37°C with shaking at 200 rpm for 6 h. The bacteria were centrifuged at 14,000 × *g* for 1 min, and the supernatant was discarded. TRIzol Reagent (1 mL) was added to the cultures in an ice bath for 30 min, and then 200 μL of chloroform was added. After the mixture was centrifuged at 14,000 × *g* at 4°C for 30 min, the aqueous phase was transferred to a new tube containing an equal volume of isopropyl alcohol. The mixture was centrifuged again at 14,000 × *g* at 4°C for 30 min, and the supernatant was precipitated with 1 mL of precooled 75% ethanol, air-dried in a biosafety cabinet for 5 min, and dissolved in 50 μL diethyl pyrocarbonate (DEPC)-treated water. A NanoDrop^™^ 2000 spectrophotometer was used to determine the concentration and purity of the extracted RNA; agarose gel electrophoresis was used to confirm the integrity of the RNA; and an Agilent 2100 Bioanalyzer was used to determine the RNA integrity number (RIN). Ribosomal RNA was removed by the RIbo-Zero kit to enrich mRNA. Then add a fragment buffer at an appropriate temperature to the enriched RNA to break the RNA into small fragments. Using the fragmented RNA as a template, first-strand cDNA was generated using the random hexamer-primed reverse transcription., and then add the buffer, dNTPs (dTTP in dNTP is replaced by dUTP), the DNA polymerase I and RNase H to synthesize the two-strand of cDNA. AMPure XP beads were then used to purify double-stranded cDNA and then the USER enzyme was used to degrade the second strand of cDNA containing U. Afterwards, A-Tailing Mix and RNA Index Adapters was added by incubating to end cDNA repair. Finally, the cDNA fragments obtained from previous steps were amplified by PCR, and products were purified by Ampure XP Bead, to get the final library. After the library was constructed, Qubit 2.0 was used for preliminary quantification. Then Agilent 2100 BioAnalyzer was used to detect the insert size of the library. Q-pcr was used to accurately quantify the effective concentration of the library to ensure the quality of the library. The raw reads generated in this study have been deposited in the Sequence Read Archive database of the National Center for Biotechnology Information (NCBI).

### Detection of differentially expressed genes (DEGs)

The SOAPnuke software (v2.1.0) was used to filter the raw reads and obtain clean reads. The main parameters were-lowQual = 20, -nRate = 0.005, -qualRate = 0.5, and all other parameters were the default parameters. Using SOAP2.2.1, the second-generation sequence of each sample was compared, after quality control, with the reference genome and the reference transcript sequence (of the strain DP2019 genome). The input data for the gene differential expression analysis was expected from the gene expression level analysis_ Count data. The R language package edgeR was used for the different analysis. The screening threshold was FDR < 0.05 and log_2_FC (fold change (condition 2/condition 1) for a gene) > 1 or log_2_FC < −1. The GO enrichment analysis used a hypergeometric distribution. As in the KEGG enrichment analysis, we selected GO terms with FDR ≤ 0.05 as significantly enriched GO entries. The R software was used for the pathway enrichment analysis.

### Quantitative real-time PCR

Bacteria were harvested when OD_600_ = 1.0 and their RNA was extracted with RNAiso Plus, according to the manufacturer’s instructions (TaKaRa). The first-strand cDNA was reverse transcribed from 1 μg of total RNA with FastKing gDNA Dispelling RT SuperMix (Tiangen). Quantitative real-time PCR (qPCR) was performed in the CFX96 Touch Real-Time PCR Detection System (Bio-Rad, United States) with TB Green^®^ Premix Ex Taq™ II (Tli RNaseH Plus; TaKaRa). The primers used are shown in Table 1. To standardize the results, the relative abundance of *gyrB* mRNA was used as the internal standard.

**Table 1:** Sequences of the primers used for RT–qPCR validation in this study.

Gene	Primer	Sequence
*gyrB*	1-Forward	AATCACGTCGTGGTCTTGCT
	2-Reverse	ACGACGAGGTTCAACAGCAT
*flhB*	1-Forward	TAAACTGGCAAAACGGGGGA
	2-Reverse	TGTGGATGAGTGGGCGTTTT
*hyfB*	1-Forward	CGGCGTGGATAGATTCGTCA
	2-Reverse	GGCGCATGGAGTATGGGATT
*hyfC*	1-Forward	CACGACCAGCAATTTCACGG
	2-Reverse	CTGAGCTGTTCCTTGCCGTA
*fdhF1*	1-Forward	CACCTATGATCCGCCGTGAA
	2-Reverse	CGATAACCGCACGAGCAAAT
*fdhF2*	1-Forward	TACCAATGGTGGATTGGGGC
	2-Reverse	GCCTTCAGCGGCTTCTCTTA
*fdhF3*	1-Forward	TGAGCGACAAGAGTAGTGCC
	2-Reverse	ATGGCCTTGTCCTGAACTCG
*mrpA*	1-Forward	GGTGGCCGTAGTAATTCTCGT
	2-Reverse	ACCCGCATCGGTGATAACAA
*fimA*	1-Forward	GCAGCAACCAATGTAGCGTT
	2-Reverse	GTAACGTTTGCGCTACCTGC
*fimC*	1-Forward	GTCCTAGTGGATTGTCCGCTT
	2-Reverse	GTCGGCTGCGGCTAAATTG
*fimF*	1-Forward	ACGCCTTACTCGCCATTGAT
	2-Reverse	TCCGGCTTGAACAGGAAGTT
*f17bG*	1-Forward	CGTGGTGTATGGCCCTGATA
	2-Reverse	CCCGGTTTTAGTGTCCCAGT
*papA*	1-Forward	TGGTTAGCACTTTCGGTAGCA
	2-Reverse	TGGCTTTTTCTGCGACTGTT

### Statistical analysis

Statistical significance was assessed with analysis of variance (ANOVA) in GraphPad Prism 6.0. All data were analyzed with one-way or two-way ANOVA. *P* < 0.05 indicated statistically significant differences.

## Data availability statement

The data presented in the study are deposited in the NCBI repository under accession numbers PRJNA953519 and CP110673.

## Ethics statement

The animal study was reviewed and approved by the Laboratory Animal Welfare and Ethics Committee of the Institute of Testing and Analysis, Guangdong Academy of Sciences (Guangzhou Analysis and Testing Center, China), protocol number W220022.

## Author contributions

XDe, LP, and YH: conceptualization and methodology. YH: data curation, formal analysis, and writing – original draft preparation. XDe and LP: funding acquisition and project administration. YH, NH, and XDi: investigation and validation. XDe, LP, and QJ: resources. XDe, NH, QJ, and XDi: supervision. YH and JC: writing – review and editing. All authors contributed to the article and approved the submitted version.

## Funding

This work was supported by the Guangdong Natural Science Foundation (grant 2020A1515011432). The funders had no role in study design, data collection and analysis, decision to publish, or preparation of the manuscript.

## Conflict of interest

The authors declare that the research was conducted in the absence of any commercial or financial relationships that could be construed as a potential conflict of interest.

## Publisher’s note

All claims expressed in this article are solely those of the authors and do not necessarily represent those of their affiliated organizations, or those of the publisher, the editors and the reviewers. Any product that may be evaluated in this article, or claim that may be made by its manufacturer, is not guaranteed or endorsed by the publisher.

## Supplementary material

SUPPLEMENTARY FIGURE S1 Flagellar synthesis pathway (Aizawa, 2001; Aldridge and Hughes, 2002; Liu and Ochman, 2007; Morimoto and Minamino, 2014; Osterman et al., 2015; Khan et al., 2020; Takekawa et al., 2020). The blue box represents the up-regulation of DEGs.
